# Data on restaurant tipping from a Norwegian survey experiment

**DOI:** 10.1016/j.dib.2020.106441

**Published:** 2020-10-21

**Authors:** Christer Thrane, Erik Haugom

**Affiliations:** Inland Norway University of Applied Sciences, Inland School of Business and Social Sciences

**Keywords:** Tipping amount, Tipping behaviour, Peer effects, Bill size, Service quality, Tipping percent, Tipping probability, Stiffing

## Abstract

The data are related to the research article “Peer effects on restaurant tipping in Norway: An experimental approach [1]”. The data stem from a survey experiment performed on students attending the Inland Norway University of Applied Sciences in December 2019 and pertain to 709 observations. The key variables are (1) tip amount, (2) peer tipping, (3) bill size, and (4) service quality. The data also contain information on background variables such as previous experience from the service industry, financial situation of respondents, gender, and age. We display the raw data organized in such way that they can be easily downloaded and used directly to either (1) replicate the analyses performed in the related research article, or (2) to run one's own analyses on the topic of interest. The data may also be useful to lecturers teaching students about the key concepts of survey experiments and causal modelling.

## Specifications Table

**Table:** 

Subject	Social Sciences (General)
Specific subject area	Business Economics with a special focus on the restaurant industry and tipping practices.
Type of data	Table in a Worksheet
How data were acquired	Survey experiment among students at Inland Norway University of Applied Sciences using 12 different versions of the same questionnaire type. The questionnaire is provided as a supplementary file.
Data format	Raw data and calculated variables needed to replicate analyses in related research article.
Parameters for data collection	At least 50 observations for each of the 12 experiment groups.
Description of data collection	The data was collected in a survey where we combined (1) online-recruitment on the University's learning management system with (2) on-campus recruitment where students were asked directly to participate via an iPad.
Data source location	Institution: Inland Norway University of Applied Sciences City/Town/Region: Lillehammer/Inland County Country: Norway
Data accessibility	With the article
Related research article	Christer Thrane and Erik Haugom, Peer effects on restaurant tipping in Norway: An experimental approach, Journal of Economic Behavior & Organization, 176, August 2020, pp. 244–252, https://doi.org/10.1016/j.jebo.2020.04.010

## Value of the Data

• Data on tipping behaviour traditionally stem from regular surveys, exit-surveys, or transaction data from the restaurant. The data offered here use an experimental design and contain information on tip amount for various levels of service quality, bill size, and peer tip amount.
• Academic researchers and analysts working in the restaurant/service industry can benefit from these data to get new insight on what causes variation in tipping behaviour and particularly to examine the effects from peers tipping behaviour. Lecturers may also benefit from using these data when teaching students the key concepts of survey experiments and causal modelling in general.
• The data may be used to gain further insight by replicating and extending the analyses performed and reported in the related research article. For example, it is possible to include more explanatory variables, transform some of those already used into new variables, create interaction-variables, and more. The data and survey experiment in general may also be used as a starting point when other scholars want to create their own experiments.
• The data are collected in Norway where diners are recognized by a high stiffing probability. This makes the data unique in a tipping context and adds additional value.

## 1 Data Description

The data stem from a survey experiment carried out among students of a large Norwegian university college. The questionnaire is provided as a supplementary file. In Table 1 we include information about the various items included in the data table accompanying this article. The table includes information about the values the various variables can assume and the mean/standard deviation. Please note that we use a “-“ to indicate that the mean and standard error do not make sense.

**Table 1:** Description of data items accompanying the article.

Variable	Mean	s.d.
Observation (1 to 709)	–	–
Questionnaire (1 to 12 – see explanation in next section)	-	-
Response Time (seconds)	148.20	276.05
Tip Amount (Norwegian Kroners. 1€ ∼ 10NOK)	24.95	22.45
Number of Restaurant Visits per Year	11.17	12.00
Experience from Service Industry (1=Yes, 0=No)	0.70	0.46
Experience from Restaurant Industry (1=Yes, 0=No)	0.29	0.45
Financial Situation (Very bad=1, Bad=2, OK=3, Good=4, Very good=5)	2.55	0.90
Academic Year (1=first year, etc.)	2.30	1.76
Type of Student (1=Full time, 0=Part time)	0.20	0.40
Gender (0=male, 1=female, 2=they)	0.31	0.48
Service (0=OK, 1=Very Good)	0.52	0.50
Bill Size (0=310, 1=510)	0.50	0.50
Peer Tips (0=0 NOK, 1=30 NOK, 2=60 NOK)	0.99	0.83
Tip in % of Bill Size	0.06	0.06
Age	27.18	9.38
Age Squared	826.79	643.45
Peer Tip of 30 NOK (0=No, 1=Yes)	0.31	0.46
Peer Tip of 60 NOK (0=No, 1=Yes)	0.34	0.47
Peer Tip Dummy Variable (0=No Peer Tip, 1=Peer tip)	0.65	0.48

## 2 Experimental Design, Materials and Methods

The data stem from a survey experiment (see [2] and [3]) carried out on students at Inland Norway University of Applied Sciences and the questionnaire is provided as a supplementary file. A survey experiment can be viewed as a *“…deliberate manipulation of the form or placement of items in a survey instrument, for purposes of inferring how public opinion works in the real world.”* [4] The item *Questionnaire* from the data description refers to the questionnaire version the respondent was given, in which the items *service (ok/very good), bill size (310/510),* and *Peer Tips (0/30/60)* were manipulated in the survey instrument. A random number generator assigned each respondent with one out of the 12 different versions of the questionnaire where the variables *service* (ok/very good), *bill size* (310/510), and *Peer Tips* (0/30/60) assumed 12 unique combinations for each of the 12 questionnaire types (2 × 2 × 3 = 12). The values the three experimental variables took on for each questionnaire type may be read directly from the data file and are summarized in Table 2. The table shows that each level of the various items occurs in a certain fraction of all the questionnaires. For the items *service* and *bill size* the two levels (ok/very good and 310/510) occur in 6 out of 12 questionnaires each. The levels of the item *Peer Tip* (0/30/60) occur in 4 out of 12 questionnaires. The way the manipulation is set up ensures that all combinations of the three key items (*service, bill size, peer tips*) are covered.

**Table 2:** Illustration of how the 12 questionnaires varies across key experiment variables.

Questionnaire # (1 to 12)	Service (0=OK, 1=Very Good)	Bill Size (0=310, 1=510)	Peer Tips (0=0 NOK, 1=30 NOK, 2=60 NOK)
1	0	0	0
2	1	0	0
3	0	0	1
4	1	0	1
5	0	0	2
6	1	0	2
7	0	1	0
8	1	1	0
9	0	1	1
10	1	1	1
11	0	1	2
12	1	1	2

The data file accompanying this article consists of the raw coded data points as they were provided by the respondents in the survey experiment. For the benefit of those wanting to replicate the analyses presented in the related research article we have calculated the variables needed to do so from the raw data. These are: (1) Tip in % of Bill Size (TipAmount/Billsize), (2) Age Squared (Age×Age), (3) Peer Tip of 30 NOK (1 if Peer TiP = 30, 0 otherwise), (4) Peer Tip of 60 NOK (1 if Peer Tip of 60, 0 otherwise), (5) Peer Tip Dummy Variable (1 if Peer Tip, 0 otherwise).

## Ethics Statement

All respondents participating in the survey experiment provided their informed consent before participating.

## Declaration of Competing Interest

None.

## Appendix Supplementary materials

Image, application 1

Image, application 2

## References

[1] Thrane, C. & Haugom, E. (2020). Peer effects on restaurant tipping in Norway: an experimental approach, J. Econ. Behav. Organ., 176, August 2020, pp. 244–252.

[2] Mutz D.C. (2011). Population-Based Survey Experiments.

[3] S.L. Nock, & T.M. Guterbock, Survey experiments, in P. V. Marsden & J. D. Wright (Eds.), Handbook of Survey Research. Second Edition, Emerald, United Kingdom pp. 837–864.

[4] Gaines B.J., Kuklinski J.H., Quirk P.J. (2007). The logic of the survey experiment reexamined. Political Anal..

