# Supplementary material for: Data on restaurant tipping from a Norwegian survey experiment
Source: Data Brief. 2020 Oct 21;33:106441. doi: 10.1016/j.dib.2020.106441 (PMC7599430; doi:10.1016/j.dib.2020.106441)
Supplement: Supplementary file 2 [file mmc2.docx]

**Questionnaire: Data on restaurant tipping from a Norwegian survey experiment**

1. You are having dinner at a restaurant together with friends a Saturday evening, and you are about to pay the bill. You are *only* going to pay for *your own* meal/drinks. Are you able to imagine this scenario?

Yes, continue____________ No, can therefore not continue the survey _________

2. **Read carefully!** The meal tasted good and your personal bill amounts to 310 NOK. The service was OK, and the person sitting next to you, who had eaten/drunk approximately the same as you, gives 0 NOK in tip. How much do you tip in this situation? _______________ (write a number, 0 NOK is a possible answer.)

3. How many times have you dined at a restaurant the last year (do not count fast-food restaurant visits)?

______ times (Write a number, 0 is a possible answer.)

4. Do you have work experience from the service industry? No _____ Yes, some _____ Yes, much______

5. Do you have work experience from the restaurant business? No _____ Yes, some _____ Yes, much ____

6. How is your financial situation compared to a typical student? Very bad___ Bad___ OK ___ Good ___ Very good ___

7. What year of study are currently at? (write a number; 1 is first year of study, 2 is the second year of study, and so forth).

8. Are you studying full time or part time? Full time _____ Part time _____

9. Campus location? 8 alternatives

10 Gender? _____ Female _____ Male _____ Other

11. Age? _____ Years (Write a number.)

Thank you very much!

Question 2 is randomized in 12 variants in the following way:

Bill size: 310 NOK; 510 NOK (two levels)

Service level: OK; Very good (two levels)

Tip amount: 0 NOK; 30 NOK; 60 NOK (three levels)
